# Findings on Thoracic Computed Tomography Scans and Respiratory Outcomes in Persons with and without Chronic Obstructive Pulmonary Disease: A Population-Based Cohort Study

**DOI:** 10.1371/journal.pone.0166745

**Published:** 2016-11-18

**Authors:** Wan C. Tan, Cameron J. Hague, Jonathon Leipsic, Jean Bourbeau, Liyun Zheng, Pei Z. Li, Don D. Sin, Harvey O. Coxson, Miranda Kirby, James C. Hogg, Rekha Raju, Jeremy Road, Denis E. O’Donnell, Francois Maltais, Paul Hernandez, Robert Cowie, Kenneth R. Chapman, Darcy D. Marciniuk, J. Mark FitzGerald, Shawn D. Aaron, Canadian Respiratory Research Network and the CanCOLD Collaborative Research group

**Affiliations:** 1 Center for Heart Lung Innovation, St. Paul's Hospital, University of British Columbia, Vancouver, BC, Canada; 2 Department of Radiology, St. Paul's Hospital, University of British Columbia, Vancouver, BC, Canada; 3 Respiratory Epidemiology and Clinical Research Unit, Montreal Chest Institute, McGill University, Montréal, QC, Canada; 4 University of British Columbia, Vancouver General Hospital, Institute for Heart and Lung Health, Vancouver, BC, Canada; 5 Division of Respiratory & Critical Care Medicine, Queen’s University, Kingston, ON, Canada; 6 Hospital Laval, Centre de Pneumologie, Institute Universitaire de Cardiologie et de Pneumologie de Quebec, Universite Laval, Quebec, QC, Canada; 7 Division of Respirology, QEII Health Sciences Centre, Dalhousie University, Halifax, Nova Scotia, Canada; 8 Departments of Medicine and Community Health Sciences, University of Calgary, Calgary, Alberta, Canada; 9 Asthma & Airway Centre, University Health Network, Toronto, ON, Canada; 10 Division of Respirology, Critical Care and Sleep Medicine, and Airway research Group, University of Saskatchewan, Saskatoon, SK, Canada; 11 Ottawa Hospital Research Institute, University of Ottawa, Ottawa, ON, Canada; National and Kapodistrian University of Athens, SWITZERLAND

## Abstract

**Background:**

Thoracic computed tomography (CT) scans are widely performed in clinical practice, often leading to detection of airway or parenchymal abnormalities in asymptomatic or minimally symptomatic individuals. However, clinical relevance of CT abnormalities is uncertain in the general population.

**Methods:**

We evaluated data from 1361 participants aged ≥40 years from a Canadian prospective cohort comprising 408 healthy never-smokers, 502 healthy ever-smokers, and 451 individuals with spirometric evidence of chronic obstructive pulmonary disease (COPD) who had thoracic CT scans. CT images of subjects were visually scored for respiratory bronchiolitis(RB), emphysema(E), bronchial-wall thickening(BWT), expiratory air-trapping(AT), and bronchiectasis(B). Multivariable logistic regression models were used to assess associations of CT features with respiratory symptoms, dyspnea, health status as determined by COPD assessment test, and risk of clinically significant exacerbations during 12 months follow-up.

**Results:**

About 11% of life-time never-smokers demonstrated emphysema on CT scans. Prevalence increased to 30% among smokers with normal lung function and 36%, 50%, and 57% among individuals with mild, moderate or severe/very severe COPD, respectively. Presence of emphysema on CT was associated with chronic cough (OR,2.11; 95%CI,1.4–3.18); chronic phlegm production (OR,1.87; 95% CI,1.27–2.76); wheeze (OR,1.61; 95% CI,1.05–2.48); dyspnoea (OR,2.90; 95% CI,1.41–5.98); CAT score≥10(OR,2.17; 95%CI,1.42–3.30) and risk of ≥2 exacerbations over 12 months (OR,2.17; 95% CI, 1.42–3.0).

**Conclusions:**

Burden of thoracic CT abnormalities is high among Canadians ≥40 years of age, including never-smokers and smokers with normal lung function. Detection of emphysema on CT scans is associated with pulmonary symptoms and increased risk of exacerbations, independent of smoking or lung function.

## Introduction

Chronic obstructive pulmonary disease (COPD) is a complex heterogeneous disease with a spectrum of overlapping clinical subtypes ultimately leading to chronic airflow limitation[1]. However, spirometry is not sensitive enough to detect early changes in lung structure or function and is not specific enough to determine underlying pathophysiologic processes responsible for airflow limitation[1–3]. Increasingly, clinical diagnosis and assessment of COPD have become multidimensional[1, 4] with the use of clinical, physiological, radiological phenotyping with multidimensional computed tomography (MDCT) scans[5] and inclusion of patient-related outcomes, including health status and exacerbation risk, in an attempt to improve assessment of the disease and its severity, thus guiding management[1, 6].

Many Canadians receive thoracic CT scans for a variety of indications, whence airway and/or parenchymal abnormalities are often detected. However, clinical relevance of these abnormalities, especially in never-smokers and those with normal lung function, is unknown. The primary aim of this study was to: 1) ascertain the prevalence of emphysema and airway abnormalities (e.g. bronchiolitis, bronchiectasis, etc) in the general Canadian population ≥40 years of age, including never-smokers and those with normal lung function; 2) determine the relationship of detected CT abnormalities with pulmonary symptoms, health status, and clinical outcomes, such as risk of exacerbations in the general population.

## Materials and Methods

### Study population

Methodology of the prospective Canadian Cohort of Obstructive Lung Disease (CanCOLD) observational study (ClinicalTrials.gov:NCT00920348) has been reported previously[7]. Briefly, we enrolled subjects from a core sample of 6,592 persons randomly recruited from 9 sites across Canada[7–9]. Participants included individuals ≥40 years who were: i) healthy persons who never smoked (never-smokers) (≤1/20 pack year of tobacco-smoking history, FEV_1_/FVC**≥**5^th^ percentile [LLN]); ii): smokers (ever-smokers) with post-bronchodilator FEV_1_/FVC**≥**LLN; iii) mild COPD (post-bronchodilator FEV_1_/FVC**<** LLN & FEV_1_pred≥80%); iv) moderate COPD (FEV_1_/FVC**<** LLN & 50%≤FEV_1_pred<80%); and v) severe to very severe COPD (FEV_1_/FVC**<** LLN & FEV_1_pred<50%)[10, 11].

The study was approved by the respective university and institutional ethical review boards at each participating site. All participants gave written informed consent.

### Methods

We obtained data which included responses to interviewer-administered questionnaires on smoking and occupational exposures, respiratory symptoms and comorbidties, spirometry measurements made before and 15 minutes after inhalation of 200mcg albuterol, full lung function measurements, thoracic CT scans, and 1 year prospective follow-up data on exacerbation-like respiratory events (see below for definition) captured via 3-monthly telephone administered questionnaires[12].

### Computed tomographic lung scans

Scanning was performed using a standard low dose protocol without bronchodilation within one day of lung function testing[13]. All CT scans were acquired using multidetector-row CT (MDCT) scanners with a minimum of 16 rows at suspended full inspiration without administration of intravenous contrast (details in S1 Text).

### CT image analysis

Images 1 mm thick were assessed and graded by two thoracic radiologists with >10 years experience in chest CTs, who were blinded to the characteristics and group assignment of participants. Bronchiolitis was defined as ill-defined centrilobular micronodules (S1 Fig)[5, 14–16] and graded based on the quartile system according to the following scale: none = 0, trivial = 1, mild = 2, moderate = 3, and severe = 4[16, 17]. Study definition for the presence of respiratory bronchiolitis was a score of ≥2.

For grading emphysema, each lung was divided into 6 zones [(upper-left and upper-right above the carina; mid (middle-left and middle-right) between carina and inferior pulmonary veins; and lower (lower-left and lower-right) zones]. The extent of zonal emphysema was scored on a 5 point scale as follows: 0 = no emphysema, 1 = 1–25% (trivial), 2 = 26–50% (mild), 3 = 51–75% (moderate), 4 = 76–100% (severe-very severe) (S2 Fig)[14]. Presence of emphysema was a summation emphysema score of ≥1. Presence of expiratory air-trapping, bronchial wall thickening, and bronchiectasis were assessed based on morphological criteria from the Fleishner glossary of terms for thoracic imaging[15].

We initially evaluated inter-observer agreement in a subset of 50 subjects. As the weighted kappa scores for all variables were comparable to or higher than previously reported[14, 18], the remaining scans were read randomly and singly by one of the two radiologists (details in S1 Text).

### Patient-reported outcomes

Outcomes of interest here consisted of respiratory symptoms including chronic cough, chronic phlegm, wheeze, dyspnea scale≥2 according to the modified Medical Research Council (mMRC) scale[19], CAT score≥10[20] and exacerbation frequency ≥2 in the following 12 months[12, 21]. Chronic cough and chronic phlegm were defined as cough/phlegm on most days for at least 3 months in two consecutive years[8, 11]. Wheeze was defined as wheezing in the chest at any time in the last 12 months[8, 11]. Exacerbation data were collected prospectively through subject telephone interviews conducted tri-monthly; only subjects completing the 12 months follow-up were included. An episode of exacerbation was defined as increased dyspnea, sputum volume, or sputum purulence for at least 2 days that might have affected work, and/or required utilization of antibiotics, corticosteroids, doctor visits, emergency room visits, or hospitalizations[1, 11, 22].

### Statistical Analyses

All analyses were performed using SAS V.9.4 (SAS Institute, Cary, North Carolina, USA).

Descriptive statistics are shown as counts and percentages for categorical data, and means and standard deviations for continuous variables, unless otherwise stated. Non-parametric Kruskall-Wallis test was used to compare continuous variables with post-hoc comparisons using Mann-Whitney test, and chi-squared test with CompProp procedure for comparison of proportions. A two-sided p<0.05 was considered statistically significant. For multiple comparisons, p values were adjusted by Holm-Bonferroni correction.

Sensisitivity analyses were performed: 1) to address the effect of a more stringent threshold for emphysema, and the caveat of regional heterogeneity of emphysema in the lung, we conducted sensitivity testing by repeating analyses for the emphysema threshold emphysema score>2; and different definitions (mean score, maximum score). 2) To address confounders caused by asthma and restrictive diseases in COPD and nonCOPD subgroups respectively, we repeated the prevalence of all CT abnormalities after excluding: a) those with a history of asthma (confounder for COPD); and b) those with preserved ratio, low FEV_1_ and low FVC (restrictive physiology, confounder for non-COPD)[23].

To address the association of CT parameters (independent variable) with patient-reported outcomes (dependent variables), multivariable logistic regression models were constructed using each of the CT variables separately with adjustment for age, sex, BMI, pack years, and FEV_1_.

## Results

We evaluated baseline data of 1361 participants, aged ≥40 years who had thoracic CT scans. The study cohort consisted of 408 never-smokers with normal lung function, 502 ever-smokers with normal lung function, and 451 individuals with COPD of different grades of severity.

### Patient characteristics

Table 1 shows baseline demographic characteristics: smoking status, pack years, history of asthma, and spirometry and lung physiological measurements for the whole cohort, stratified into 5 study subgroups. There was no difference in demographic characteristics, occupational exposures, or asthma history between never-smokers and ever-smokers with normal lung function. Subjects with mild COPD were younger than all other subgroups (all p<0.0003). Moderate-to-severe COPD comprised more current smokers with greater pack-year consumption of tobacco compared to mild COPD (all p< 0.0025). A self-reported history of asthma was more frequent in COPD subgroups compared to non-COPD individuals (all p< 0.001). Pulmonary physiological measurements discriminated COPD of all severity from never-smokers and ever-smokers without COPD (all p< 0.01), although smokers without COPD had slightly increased residual volume (RV) and functional residual capacity (FRC) compared with never-smokers (p<0.001, 0.0042).

**Table 1:** Demographics, exposures, Clinical Characteristics, Pulmonary function and CT measurements of the study population stratified into five subgroups according to post bronchodilator spirometry (N = 1361).

	‡ Normal	‡ At Risk	‡ LLN Mild	‡ LLN Moderate	‡ LLN Severe/ V Severe
	N = 408(30%)	N = 502(37%)	N = 198(15%)	N = 216(16%)	N = 37(3%)
*Demographics*					
Age, Years, mean(SD)	68.0(9.4)	67.7(9.0)	62.8(11)*^#^	65.3(10.5)	69.0(9.1)^ϕ^
Sex, female, n(%)	184(45)	187(37)	100(51)^#^	109(50)^#^	20(54)
BMI, kg/m^2^, mean(SD)	27.5(5.6)	28.0(4.9)	27.2(5.1)	27.8(5.4)	29.1(6.1)
*Exposures*					
Ever Smoker, n(%)	0	502(100)	133(67)^#^	163(75)^#^	33(89)^#^^ϕ^
Current Smoker, n(%)	0	104(21)	30(15)	54(25)	14(38)^ϕ^
Pack Years of Cigarettes, mean(SD)	0	24.0(23.4)	18.8(22.3)^#^	27.3(27.2)^ϕ^	36.2(30.8)^ϕ^
Asthma, n(%)	20(5)	38(8)	34(17)*^#^	45(21)*^#^	12(32)*^#^
Organic/Inorganic Dust/Gas/Vapour, n(%)	45(11)	46(9)	19(10)	29(13)	8(22)
*Spirometry Results*, *mean (SD)*					
% Predicted FEV_1_	100.1(17.4)	96.0(17.2)*	92.1(10.9) *	69.2(11.6)*^#^^ϕ^	46.6(9.7)*^#^^ϕ^^θ^
% Predicted FVC	101.0(17.4)	98.6(16.8)	108.9(13) *^#^	90.5(13.6)*^#^^ϕ^	76.0(14.9)*^#^^ϕ^^θ^
Max post-BD FEV_1_/FVC	75.0(6.9)	72.9(6.8)*	64.6(7.3) *^#^	58.2(9.1) *^#^^ϕ^	47.0(10.6)*^#^^ϕ^
*PFT Results*, *mean (SD)*					
DLCO, ml/min/mmHg	21.8(6.6)	21.6(6.4)	21.4(6.8)	19.7(7.6)*^#^^ϕ^	13.9(4.5)*^#^^ϕ^^θ^
DLCO, % Predicted	112.2(23.3)	108.7(28.8)	105.0(24.0)*	97.3(25.9)*^#^^ϕ^	74.9(19.8)*^#^^ϕ^^θ^
FRC, L	3.2(0.9)	3.4(0.9)*	3.6(0.9)*^#^	3.8(1.1)*^#^	4.2(1.0)*^#^^ϕ^
FRC, % Predicted	104.4(24.2)	106.1(22.2)	116.6(23.4)*	121.1(30.0)*^#^^ϕ^	141.2(29.8)*^#^^ϕ^^θ^
RV, L	2.3(0.6)	2.4(0.7)*	2.6(0.8)*	2.9(0.9)*^#^^ϕ^	3.5(0.9)*^#^^ϕ^^θ^
RV, % Predicted	109.7(27.4)	113.8(28.4)	125.7(33.8)*^#^	144.0(39.5)*^#^^ϕ^	175.6(42.1)*^#^^ϕ^^θ^
TLC, L	6.1(1.4)	6.3(1.4)	6.7(1.4)*	6.3(1.5)	6.1(1.2)
TLC, % Predicted	116.1(18.3)	115.9(16.6)	125.7(14.5)*^#^	119.3(18.1)^ϕ^	122.5(19.5)
ERV, L	1.0(0.6)	1.0(0.6)	1.1(0.6)	0.8(0.5)*^#^^ϕ^	0.7(0.3)^#^^ϕ^
RV/TLC	0.4(0.1)	0.4(0.1)	0.4(0.1)	0.5(0.1)*^#^^ϕ^	0.6(0.1)*^#^^ϕ^^θ^
*CT Features*, *n(%)*					
Bronchiolitis	48(11.8)	82(16.3)	18(9.1)^#^	20(9.3)^#^	4(10.8)
Emphysema	45(11.0)	149(29.7)*	71(35.9)*	108(50.0) *^#^^ϕ^	21(56.76) *^#^
Bronchial wall thickening	127(31.1)	289(57.6)*	126(63.6)*	169(78.2) *^#^^ϕ^	35(94.6)*^#^^ϕ^^θ^
Expiratory air trapping	102(25.0)	176(35.1)*	50(25.3)	51(23.6)^#^	10(27.0)
Bronchiectasis	81(19.9)	100(19.9)	28(14.1)	48(22.2)	13(35.1)^ϕ^

Data are mean (SD) or count (%).

^**‡**^ Normal = Never smoker with no obstruction (FEV1/FVC≥LLN); At Risk = Ever smoker with no obstruction (FEV1/FVC≥LLN); COPD: LLN-mild = Post FEV_1_/FVC<LLN and FEV_1_% Pred≥80%; LLN-moderate = Post FEV_1_/FVC<LLN and 50%≤FEV_1_% Pred<80%; LLN-severe/v severe = Post FEV_1_/FVC<LLN and FEV_1_% Pred<50%. Max post-BD = maximal post bronchodilator.

* significantly different to Normal (reference);

^#^ significantly different to ‘At Risk’ (reference);

^ϕ^ significantly different to LLN-mild (reference);

^θ^ significantly different to LLN-moderate (reference).

### Prevalence of CT features by subgroups

**Fig 1:**
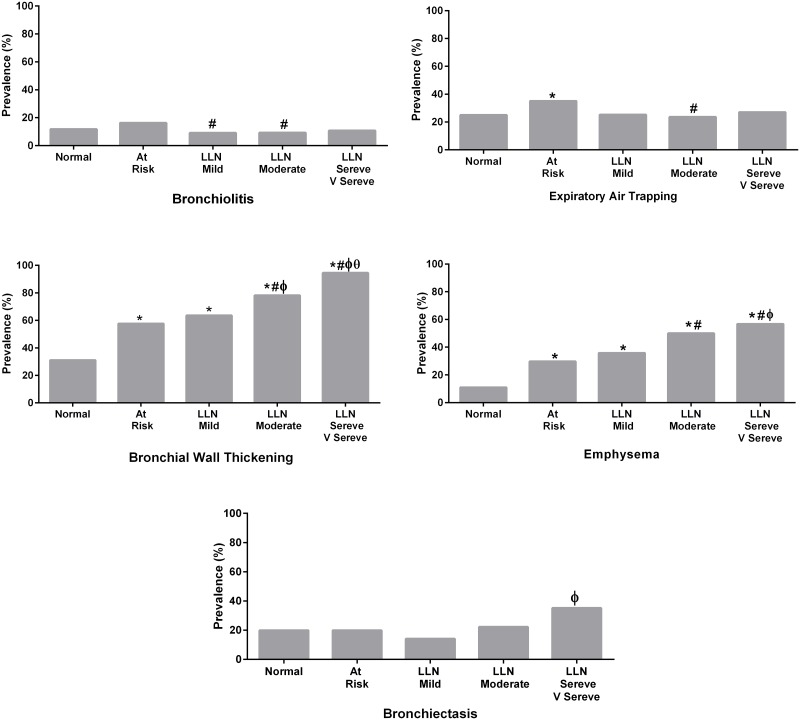
Prevalence of respiratory bronchiolitis; air-trapping; bronchial wall thickening; emphysema; bronchiectasis. Five study subgroups are: Normal (FEV_1_/FVC ≥ LLN and never smoker); At Risk (FEV_1_/FVC ≥ LLN and ever smoker); Mild COPD (FEV_1_/FVC < LLN and FEV_1_%Pred ≥ 80%); Moderate COPD (FEV_1_/FVC < LLN and 50% ≤ FEV_1_%Pred < 80%); Severe to very severe COPD (FEV_1_/FVC < LLN and FEV_1_%Pred < 50%). All P values are corrected by Holm-Bonferroni correction for multiple comparisons. P values<0.05: *ref = Normal; # Ref = At Risk; ϕ Ref = LLN Mild; θ Ref = LLN moderate.

Respiratory bronchiolitis was more frequent in ever-smokers with normal lung function (16%) compared to those with mild (9%) and moderate-severe (9%) COPD (p = 0.016, p = 0.0141). Air-trapping was more frequent in smokers without COPD (35%) compared to the normal (25%) (p = 0.0011) or moderate COPD (24%) subgroups (p = 0.0029). Prevalence of bronchial wall thickening increased in smokers without COPD (58%) compared to ‘normal’ subjects (31%) (p = 0.0003), and further increased in COPD of all severity grades (mild 64%, moderate 78%, severe 95%), (p = 0.0004, 0.0005, 0.006, respectively). Similarly, the proportion of individuals with emphysema was elevated in ever-smokers with normal lung function (30%) compared to the normal group (11%) (p = 0.0007) and stepwise increases with increasing severity of COPD (mild 36%, moderate 50%, severe 57%).Bronchiectasis prevalence was only significantly elevated in severe COPD (Table 2, Fig 1).

**Table 2:** Prevalence of CT measurements of the study population stratified into five subgroups according to post bronchodilator spirometry (N = 1361).

	‡ Normal	‡ At Risk	‡ LLN Mild	‡ LLN Moderate	‡ LLNSevere/ V severe
	N = 408(30%)	N = 502(37%)	N = 198(15%)	N = 216(16%)	N = 37(3%)
*CT Features*, *n(%)*					
Bronchiolitis	48(11.8)	82(16.3)	18(9.1)#	20(9.3)#	4(10.8)
Emphysema	45(11.0)	149(29.7)*	71(35.9)*	108(50.0)*#ϕ	21(56.76)*#
Bronchial wall thickening	127(31.1)	289(57.6)*	126(63.6)*	169(78.2)*#ϕ	35(94.6)*#ϕθ
Expiratory air trapping	102(25.0)	176(35.1)*	50(25.3)	51(23.6)#	10(27.0)
Bronchiectasis	81(19.9)	100(19.9)	28(14.1)	48(22.2)	13(35.1)ϕ

Data are mean (SD) or count (%).

^**‡**^ Normal = Never smoker with no obstruction (FEV1/FVC≥LLN); At Risk = Ever smoker with no obstruction (FEV1/FVC≥LLN); COPD: LLN-mild = Post FEV_1_/FVC<LLN and FEV_1_% Pred≥80%; LLN-moderate = Post FEV_1_/FVC<LLN and 50%≤FEV_1_% Pred<80%; LLN-severe/v severe = Post FEV_1_/FVC<LLN and FEV_1_% Pred<50%. Max post-BD = maximal post bronchodilator.

* significantly different from Normal (reference);

^#^ significantly different from ‘At Risk’ (reference);

^ϕ^ significantly different from LLN-mild (reference);

^θ^ significantly different from LLN-moderate (reference). P values were adjusted by Holm-Bonferroni correction for multiple comparisons.

#### Sensitivity tests

We consistently observed that emphysema increased in smokers, which further increased in a stepwise fashion with increasing severity of COPD, regardless of the emphysema definition used (e.g. summation score of ≥1 vs. summation score of ≥2 vs. mean score vs. maximal score). The p values for comparisons using mean score were: p = 0.0003 for smokers vs. normal; p = 0.0004 for mild COPD vs. normal; and p = 0.0005 for moderate/severe COPD vs. smokers.

When subjects with a) asthma (a self-reported history of asthma: confounder for COPD) or b) restrictive disease (subnormal FEV_1_ and FVC, but preserved FEV_1_/FVC ratio: confounder for non-COPD) [23] were excluded from the whole cohort, prevalence for each of the 5 CT features were similar to results computed using the whole cohort (S1 Table, S3 Fig; S2 Table, S4 Fig).

### Association between CT features and patient-reported outcomes

**Fig 2:**
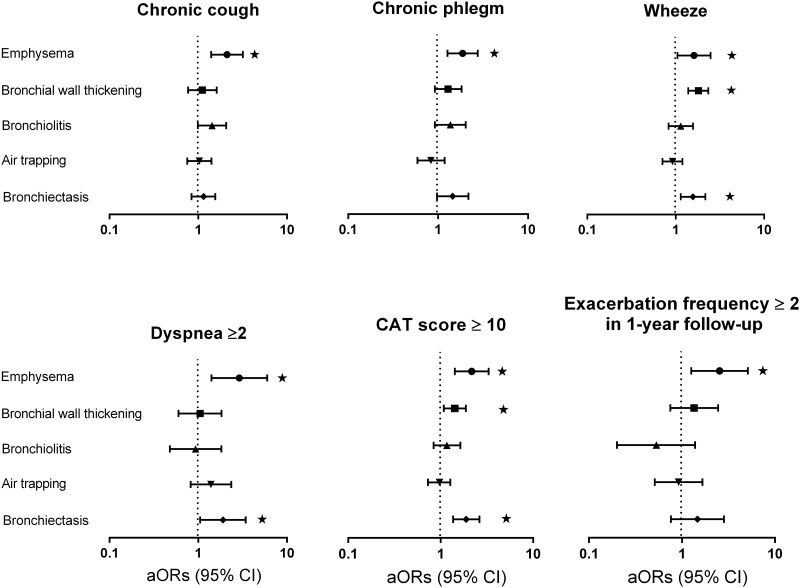
The relationship of bronchiolitis, Bronchiectasis, bronchial wall thickening, and emphysema with six Patient-Reported Outcomes. The outcomes are: chronic cough; chronic phlegm; wheezing; dyspnea [mMRC scale ≥2]; COPD assessment test [CAT] score>10; and exacerbation ≥2 in 1 year follow-up). The Odds Ratio (aOR [95% CI]) were adjusted for age, sex, BMI, Pack-years, and FEV1. * p value <0.05.

Fig 2 and Table 3 show results from multivariable logistic regression models shown as adjusted Odds Ratio, aOR(95% CI) adjusted for age, sex, BMI, pack years, and FEV_1_. Emphysema was most consistently associated with chronic cough, chronic phlegm, wheeze, dyspnea ≥2 mMRC grade, CAT score ≥10, exacerbation frequency ≥2 within the 12 month follow-up and hospitalized (severe) exacerbation frequency ≥1 within the 12 month follow-up (Table 3). Bronchial wall thickening was associated with wheeze and CAT score ≥10; bronchiectasis with wheeze, dyspnea (mMRC scale ≥2) and CAT score ≥10. No association was found between respiratory bronchiolitis or air–trapping and respiratory outcomes.

**Table 3:** The risk of visual CT variables on developing of patient-reported outcomes (data for Fig 2 in manuscript).

	Chronic Cough	Chronic Phlegm	Wheeze	Dyspnea ≥2	CAT Score ≥10	Exacerbation Frequency ≥2 in 1-year follow-up	Hospitalization Frequency for Exacerbation ≥1 in 1-year follow-up
Emphysema Score ≥ 1							
aOR	2.11	1.87	1.61	2.90	2.17	2.54	5.94
95% CI	1.40–3.18	1.27–2.76	1.05–2.48	1.41–5.98	1.42–3.30	1.26–5.11	1.32–26.73
P-value	< .001*	0.002*	0.029*	0.004*	< .001*	0.009*	0.020*
Bronchial wall thickening							
aOR	1.15	1.29	1.81	1.05	1.42	1.35	1.86
95% CI	0.84–1.56	0.92–1.82	1.39–2.34	0.60–1.83	1.08–1.87	0.75–2.45	0.38–9.15
P-value	0.392	0.141	< .001*	0.871	0.012*	0.316	0.446
Bronchiolitis Score **≥ 2**							
aOR	1.11	1.37	1.14	0.93	1.17	0.53	- #
95% CI	0.77–1.61	0.92–2.03	0.83–1.57	0.48–1.82	0.84–1.63	0.20–1.38	-
P-value	0.566	0.118	0.409	0.834	0.368	0.195	-
Air trapping							
aOR	1.03	0.83	0.92	1.39	0.97	0.92	0.63
95% CI	0.75–1.41	0.59–1.18	0.71–1.19	0.82–2.36	0.73–1.27	0.51–1.66	0.13–3.03
P-value	0.851	0.311	0.507	0.218	0.806	0.782	0.566
Bronchiectasis							
aOR	1.43	1.45	1.57	1.90	1.89	1.47	0.86
95% CI	0.99–2.06	0.97–2.17	1.14–2.17	1.05–3.42	1.36–2.63	0.76–2.83	0.16–4.58
P-value	0.056	0.069	0.006*	0.033*	< .001*	0.253	0.860

The outcomes were modeled using each of the CT variables separately with adjustment for age, sex, BMI, pack years, FEV_1_

aOR = Adjusted Odds Ratios.

* Significant association between visual CT variables and respiratory outcomes.

^#^ Due to small exposed cases, ORs were not computable. Dyspnea (MMRC scale) **≥2.**

Subgroup analyses in the ‘normal’ (never-smokers with normal lung function) and ‘at-risk’ (ever-smokers with normal lung function) showed that bronchiectasis was associated with reduced quality of life (CAT score > = 10) in both these subgroups while bronchial wall thickening was associated with chronic phlegm in ‘at-risk’ smokers. While there appeared to be a trend, there was no significant association between emphysema and clinical outcomes in these two non-COPD subgroups. (S3 and S4 Tables).

## Discussion

Eleven percent of Canadians aged ≥40 years who never smoked, and 30% of smokers with normal lung function, have evidence of emphysema on CT scans. More importantly, the presence of emphysema was related to poor outcomes as reported by patients, including chronic cough, chronic phlegm, wheeze, dyspnea, reduced health status, and increased risk of exacerbations and hospitalizations for exacerbations during 12 month follow-up.

CT emphysema and bronchial wall thickening performed best at discriminating between subjects with and without airflow limitation and between levels of severity, as previously described in patient studies. These two CT features were also increased in smokers without spirometric evidence of airway obstruction. These findings underscore the usefulness of CT features as radiological markers of COPD in ‘at risk’ smokers and in patients with mild or conceivably early COPD, and support the increasing practice of using CT-based emphysema measurements to identify patients with early COPD in clinical practice who do not demonstrate abnormal spirometry. We extend the findings of previous work, which were largely performed in selected smokers or COPD patients (e.g. ECLIPSE[13] COPDgene[14] and SPIROMICS[24] studies), by demonstrating the added value of this approach in the general population[14, 25–28]. Additionally, data could be employed to strengthen anti-smoking efforts as it added population-specific evidence to previous studies of convenient samples of selected smokers[29, 30] Another application was that radiological markers could be used to define individuals for future early interventional trials, reemphasizing the continued relevance of traditional, population-based epidemiological studies in an era of ‘mega-cohorts’ comprising administrative databases, selected participants, and consortia of cohorts[31].

The second key finding was the validation of CT features of COPD against clinically relevant health outcomes. To our knowledge, this is the first study that systematically evaluated the influence of the various discrete CT phenotypes on a wide range of clinical outcomes that matter to patients. The most clinically important CT feature was emphysema that was widely associated with symptoms, and severe dyspnea, as well as to reduced health status, and was a significant predictor of future exacerbations, including severe exacerbations requiring hospitalization. The non-significant trend of association between emphysema and outcomes in the normal and at risk non-COPD subgroups would suggest that the extent of emphysema in these two subgroups was mild. Overall, these findings underscore the role of emphysema in furthering our understanding of COPD as imaging findings of emphysema can provide information beyond FEV1 in terms of patient-related health outcomes. For instance, the presence of emphysema may tell us something about disease activity (e.g. having emphysema predicts rapid decline in lung function over time) and about poor gas exchange or gas trapping that are not captured in FEV1. CT based emphysema also provides information about heterogeneity of disease and which lobes are most affected by the disease. All of these factors may relate to patient-related symptoms and outcomes.[32]

Bronchial wall thickening and bronchiectasis were also clinically pertinent as they were related to symptoms and health status. Interestingly, presence of air trapping and bronchiolitis was not associated with any of these outcomes.

However, it is unclear why the prevalence of CT respiratory bronchiolitis and expiratory air trapping (surrogates for ‘small airways disease’) [15] was increased in smokers without COPD but not in those with established COPD, yet were not associated with clinical outcomes. A potential explanation for the lack of clinical association could be that these CT features were the earliest pathological processes occurring in the physiologically ‘silent zones’ of the lungs before more advanced destructive changes that drive airflow limitation detectable on spirometry occurred[33]. The lack of presence of bronchiolitis and air-trapping in COPD can be explained by the subsequent destruction of terminal respiratory units at sites of respiratory bronchiolitis, leaving behind changes of centrilobular emphysema in COPD [34], thus confounding the assessment of bronchiolitis and air trapping. Finally, bronchiectasis was increased in individuals with established moderate and severe COPD compared to mild COPD, suggesting it was marker of severity of disease in COPD.

### Strengths

This study involved a large prospective cohort of unselected individuals from a random sample in the general population with extensive phenotyping[7, 9], providing data that included a wide range of self-reported patient outcomes and physiological lung function measurements used for linkage to CT features from CT scans that were systematically evaluated and scored according to validated protocols by two dedicated, experienced chest radiologists. The collection of prospective exacerbation data was a definite strength. We also examined various parameters and their relationship with exacerbations requiring hospitalization. CanCOLD participants were selected randomly and not based on symptoms or “disease”, unlike previous large studies such as ECLIPSE[13], COPDgene[14], or SPIROMICS[24]. Thus, the relationship of the CT abnormalities with health status and outcomes of the subjects in the present study was likely free of ascertainment or sampling bias. Moreover, the findings here extend previously published data by demonstrating the high burden of emphysema and airway abnormalities in the general population, even among lifetime non-smokers and those with normal lung function, highlighing the importance of imaging (in addition to lung function measurements) in diagnosing early lung disease in symptomatic individuals.

### Limitations

A potential limitation in the study was that in our stratification of subject subgroups we did not exclude individuals with asthma from the analysis of chronic airflow limitation, hence some never-smokers labeled as COPD may have fixed airflow limitation and remodeling due to long-standing asthma and some may have poorly controlled asthma which was not fully reversed with bronchodilators. Finally the results here were based on analysis of the baseline CT scans assessment. Findings and changes over time would need to be confirmed by further longitudinal data.

In summary, this study defined the burden of radiological abnormalities in the lungs of the general population, from health to disease, and confirmed the clinical relevance by their associations with multiple clinical outcomes. Our study, which focused on individuals in the population, extended the generalizability of current literature on CT scans in patients to the broader population and provided fundamental data on the occurrence of structural lung changes in disease and early subclinical disease in the general population. It remains to be shown in clinical trials if these findings could be used to guide early therapy and reduce the burden of disease.

## Supporting Information

S1 Fig Noncontrast transaxial CT to represent severe respiratory bronchiolitis. (TIF)

S2 Fig Noncontrast transaxial CT image showing severe centrilobular emphysema. (TIF)

S3 Fig Prevalence of CT parameters in five study subgroups (cohort without asthma). (TIF)

S4 Fig Prevalence of CT parameters in five study subgroups (cohort without restrictive disease). (TIF)

S1 Table Prevalence of CT parameters in five study subgroups (cohort without asthma). (DOC)

S2 Table Prevalence of CT parameters in five study subgroups (cohort without restrictive disease). (DOC)

S3 Table The risk of visual CT variables on developing of patient-reported outcomes for Normal group. (DOC)

S4 Table The risk of visual CT variables on developing of patient-reported outcomes for healthy smokers (At risk) group. (DOC)

S1 Text Online text supplement. (DOC)

## Abbreviations

CT: computed tomography
CanCOLD: The Canadian Cohort of Obstructive Lung Disease
COPD: chronic obstructive respiratory disease
PB: post-bronchodilator
FEV_1_/FVC: ratio of forced expiratory volume in the first second (FEV1) / forced vital capacity (FVC)
FEV_1_/FVC**<**5^th^ percentile: FEV1/FVC ratio< 5^th^ percentile or lower limits of normal (LLN)
mMRC dyspnea scale: modified Medical Research Council dyspnea scale = scale
CAT: COPD Assessment Test
