# Supplementary figures and images for: Findings on Thoracic Computed Tomography Scans and Respiratory Outcomes in Persons with and without Chronic Obstructive Pulmonary Disease: A Population-Based Cohort Study
Source: PLoS One. 2016 Nov 18;11(11):e0166745. doi: 10.1371/journal.pone.0166745 (PMC5115801; doi:10.1371/journal.pone.0166745)

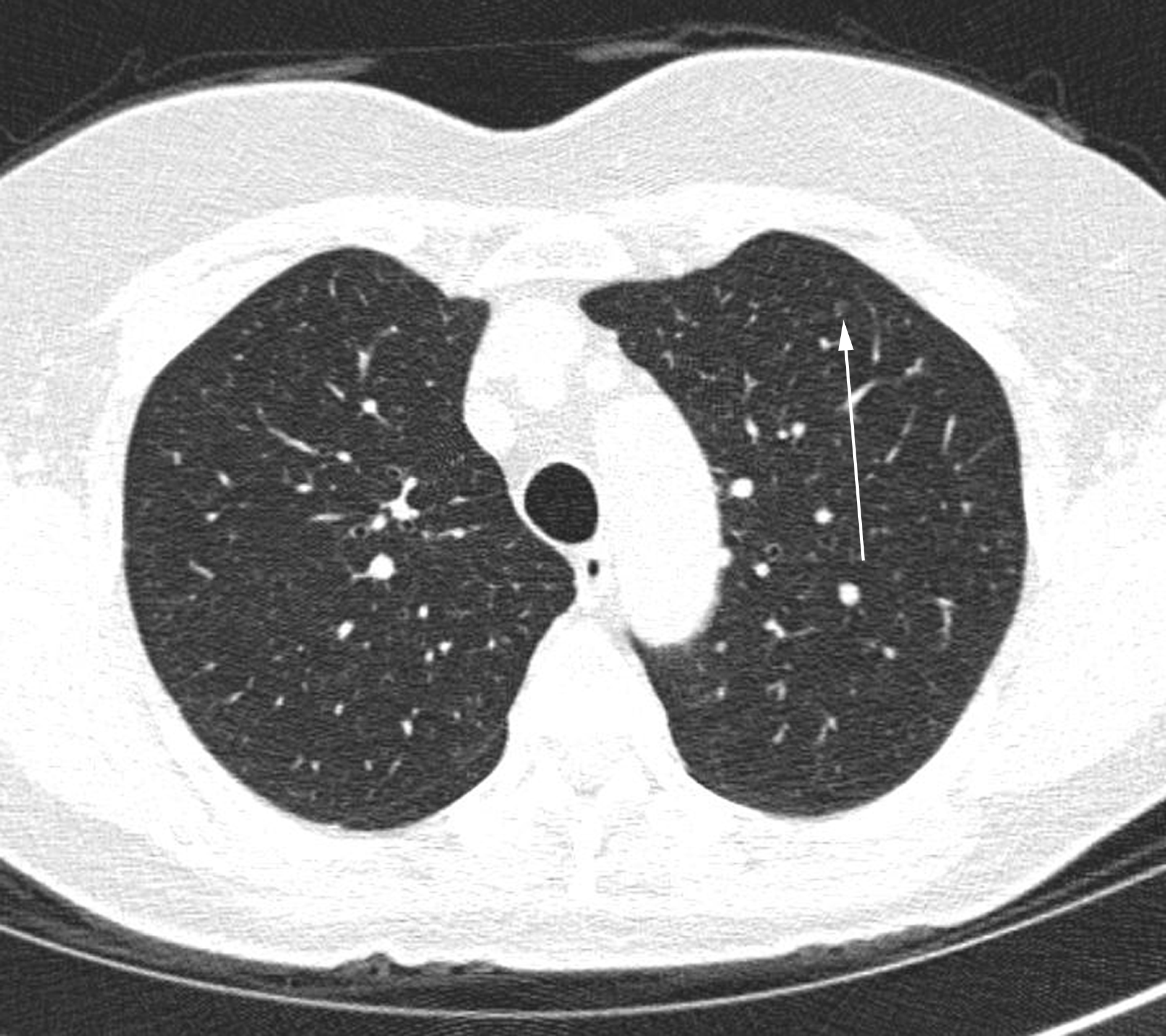

Supplement: S1 Fig — (TIF) [file pone.0166745.s001.tif]

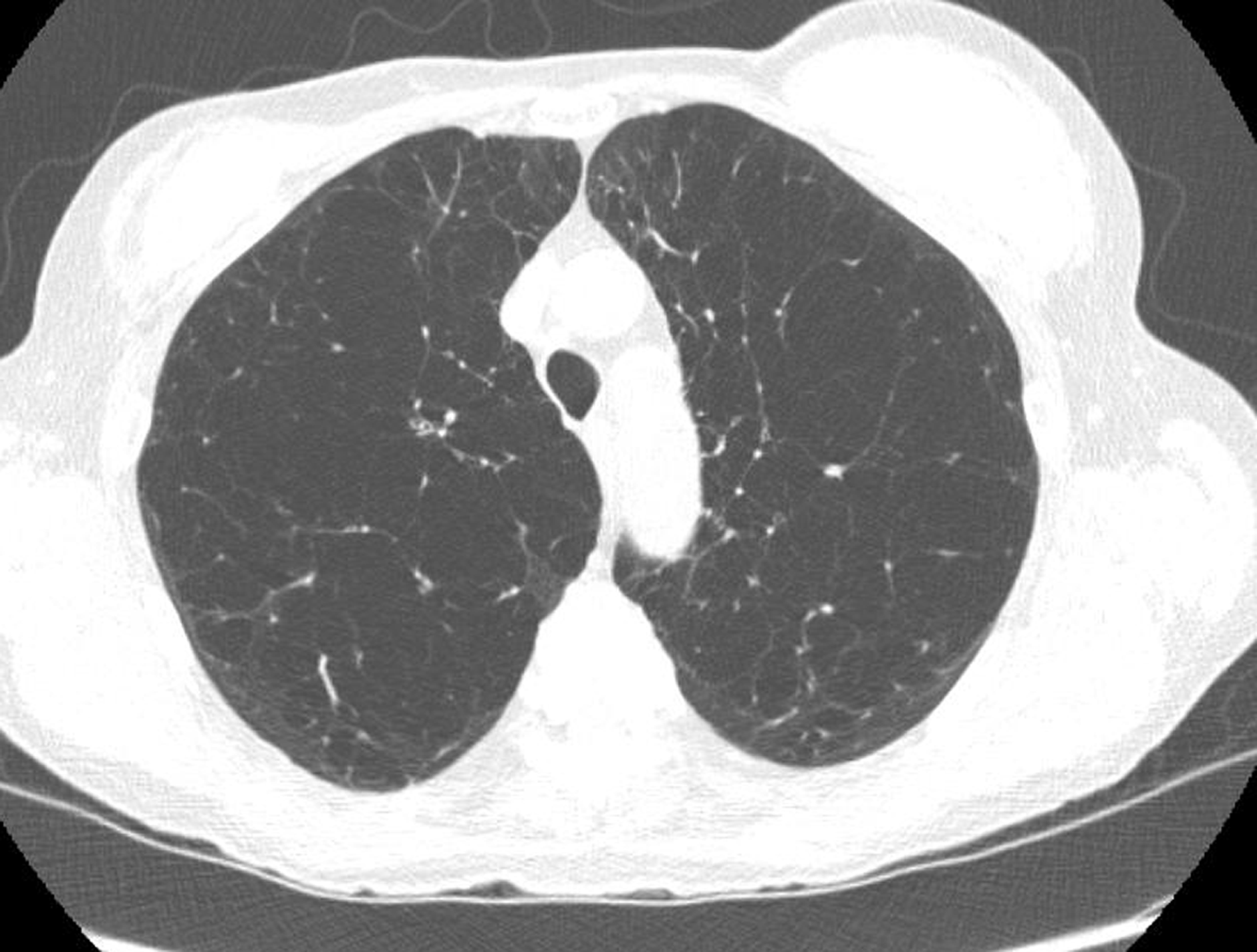

Supplement: S2 Fig — (TIF) [file pone.0166745.s002.tif]
