# Supplementary material for: Findings on Thoracic Computed Tomography Scans and Respiratory Outcomes in Persons with and without Chronic Obstructive Pulmonary Disease: A Population-Based Cohort Study
Source: PLoS One. 2016 Nov 18;11(11):e0166745. doi: 10.1371/journal.pone.0166745 (PMC5115801; doi:10.1371/journal.pone.0166745)
Supplement: S1 Table — (DOC) [file pone.0166745.s005.doc]

**Table 1** . Prevalence of CT measures for the study population stratified into five subgroups according to post bronchodilator spirometry (N=1212, subjects with history of asthma excluded)

|  | **‡** Normal | **‡** At Risk | **‡** LLN  Mild | **‡** LLN  Moderate | **‡** LLN  Server/V server |
| --- | --- | --- | --- | --- | --- |
|  | N= 388 | N= 464 | N= 164 | N= 171 | N= 25 |
| Bronchiolitis | 46(11.9) | 76(16.4) | 13(7.9)# | 13(7.6)# | 2(8.0) |
| Emphysema | 44(11.3) | 135(29.1)* | 64(39.0)* | 92(53.8)*#Ɨ | 15(60.0)*# |
| Bronchial Wall Thickening | 118(30.4) | 271(58.4)* | 105(64.0)* | 132(77.2)*#Ɨ | 23(92.0)*#Ɨ |
| Expiratory Air Trapping | 92(23.7) | 162(34.9)* | 36(22.0)# | 35(20.5)# | 7(28.0) |
| Bronchiectasis | 72(15.6) | 91(19.6) | 24(14.6) | 37(21.6) | 10(40.0)Ɨ |

Data are mean (SD) or count (%). **‡** Normal= Never smoker with no obstruction (FEV1/FVC≥LLN); At Risk=Ever smoker with no obstruction (FEV1/FVC≥LLN); LLN-Mild=Post FEV_1_/FVC<LLN and FEV_1_% Pred≥80%; LLN-Moderate= Post FEV_1_/FVC<LLN and 50%≤FEV_1_% Pred<80%; LLN- Server/V server = Post FEV_1_/FVC<LLN and FEV_1_% Pred<50%. Max post-BD = maximal post bronchodilator. Kruskal-Wallis test (without assumption of normal distribution of data) and Mann-Whitney test are used for continuous variables; χ^2^ test and CompProp procedure are used for categorical variables.* significantly different to Normal subgroup as reference; # significantly different to ‘At Risk’ subgroup as reference; Ɨ significantly different to LLN-Mild subgroup as reference; § significantly different to LLN-Moderate subgroup as reference. P values were adjusted by Holm-Bonferroni correction for multiple comparisons.
