# Supplementary material for: Findings on Thoracic Computed Tomography Scans and Respiratory Outcomes in Persons with and without Chronic Obstructive Pulmonary Disease: A Population-Based Cohort Study
Source: PLoS One. 2016 Nov 18;11(11):e0166745. doi: 10.1371/journal.pone.0166745 (PMC5115801; doi:10.1371/journal.pone.0166745)
Supplement: S2 Table — (DOC) [file pone.0166745.s006.doc]

Table S2. Prevalence of CT measures for the study population stratified into five subgroups according to post bronchodilator spirometry (N=1265, excluding subjects with low FEV1 and low FVC but preserved FEV1/FVC>LLN)

|  | **‡** Normal | **‡** At Risk | **‡** LLN  Mild | **‡** LLN  Moderate | **‡** LLN  Server/V server |
| --- | --- | --- | --- | --- | --- |
|  | **N=365** | **N=449** | **N=198** | **N=216** | **N=37** |
| Bronchiolitis | 44(12.1) | 73(16.3) | 18(9.1)# | 20(9.3)# | 4(10.8) |
| Emphysema | 42(11.5) | 134(29.8)* | 71(35.9)* | 108(50.0)*#ϕ | 21(56.8)*# |
| Bronchial Wall Thickening | 112(30.7) | 255(56.8)* | 126(63.6)* | 169(78.2)*#ϕ | 35(94.6)*#ϕ |
| Expiratory Air Trapping | 87(23.8) | 156(34.7)* | 50(25.3) | 51(23.6)# | 10(27.0) |
| Bronchiectasis | 64(17.5) | 91(20.3) | 28(14.1) | 48(22.2) | 13(35.1) ϕ |

Data are mean (SD) or count (%). **‡** Normal= Never smoker with no obstruction (FEV1/FVC≥LLN); At Risk=Ever smoker with no obstruction (FEV1/FVC≥LLN); COPD: LLN-mild=Post FEV_1_/FVC<LLN and FEV_1_% Pred≥80%; LLN-moderate= Post FEV_1_/FVC<LLN and 50%≤FEV_1_% Pred<80%; LLN-severe/v severe= Post FEV_1_/FVC<LLN and FEV_1_% Pred<50%. Max post-BD = maximal post bronchodilator.* significantly different to Normal (reference); # significantly different to ‘At Risk’ (reference) ; ϕ significantly different to LLN-mild (reference); θ significantly different to LLN-moderate (reference). P values were adjusted by Holm-Bonferroni correction for multiple comparisons.
