# Supplementary material for: Findings on Thoracic Computed Tomography Scans and Respiratory Outcomes in Persons with and without Chronic Obstructive Pulmonary Disease: A Population-Based Cohort Study
Source: PLoS One. 2016 Nov 18;11(11):e0166745. doi: 10.1371/journal.pone.0166745 (PMC5115801; doi:10.1371/journal.pone.0166745)
Supplement: S4 Table — (DOC) [file pone.0166745.s008.doc]

S4 Tab. The risk of visual CT variables on developing of #patient-reported outcomes for At risk ( ever-smokers with normal lung function) group

|  | **Chronic Cough** | **Chronic Phlegm** | **Wheeze** | **Dyspnea ≥2** | **CAT Score ≥10** | **Exacerbation Frequency ≥2 in 1-year follow-up** | **Hospitalization Frequency for Exacerbation ≥1 in 1-year follow-up** |
| --- | --- | --- | --- | --- | --- | --- | --- |
| Emphysema Score ≥ 1 |  |  |  |  |  |  |  |
| aOR | 1.05 | 1.14 | 1.14 | 0.32 | 1.28 | 1.77 | - |
| 95% CI | 0.60-1.84 | 0.62-2.09 | 0.68-1.92 | 0.07-1.51 | 0.76-2.15 | 0.68-4.62 | - |
| P-value | 0.87 | 0.678 | 0.61 | 0.15 | 0.357 | 0.242 | - |
|  |  |  |  |  |  |  |  |
| Bronchial wall thickening |  |  |  |  |  |  |  |
| aOR | 1.01 | 1.95 | 1.21 | 0.97 | 1.50 | 1.29 | - |
| 95% CI | 0.59-1.71 | 1.04-3.65 | 0.75-1.95 | 0.32-2.93 | 0.91-2.48 | 0.51-3.28 | - |
| P-value | 0.972 | 0.038* | 0.427 | 0.961 | 0.112 | 0.59 | - |
|  |  |  |  |  |  |  |  |
| Bronchiolitis Score **≥ 2** |  |  |  |  |  |  |  |
| aOR | 1.48 | 1.79 | 1.18 | 1.55 | 1.41 | 1.25 | 0.42 |
| 95% CI0.243 | 0.85-2.60 | 0.98-3.27 | 0.70-1.98 | 0.50-4.81 | 0.83-2.39 | 0.46-3.38 | 0.01-13.43 |
| P-value | 0.168 | 0.057 | 0.544 | 0.449 | 0.2 | 0.663 | 0.627 |
|  |  |  |  |  |  |  |  |
| Air trapping |  |  |  |  |  |  |  |
| aOR | 1.01 | 1.08 | 1.02 | 0.91 | 0.93 | 1.05 | - |
| 95% CI | 0.59-1.73 | 0.60-1.94 | 0.64-1.65 | 0.30-2.79 | 0.57-1.51 | 0.41-2.69 | - |
| P-value | 0.979 | 0.805 | 0.925 | 0.865 | 0.786 | 0.923 | - |
|  |  |  |  |  |  |  |  |
| Bronchiectasis |  |  |  |  |  |  |  |
| aOR | 1.25 | 1.38 | 1.62 | 1.08 | 1.81 | 0.84 | 7.01 |
| 95% CI | 0.68-2.30 | 0.70-2.71 | 0.92-2.86 | 0.28-4.17 | 1.03-3.18 | 0.26-2.68 | 0.27-184.35 |
| P-value | 0.469 | 0.349 | 0.093 | 0.909 | 0.039* | 0.77 | 0.243 |

Odds Ratios were adjusted (aOR) for age, sex, BMI, pack years, FEV_1_. * Significant association between visual CT variables and respiratory outcomes. – Due to small exposed cases, ORs were not computable. Dyspnea (MMRC scale) **≥2.**

**# Although the trends appear similar to that for the results for whole cohort, most of the CT variables were not strong predictors of patient outcomes in the subgroup analyses most likely due to a lack of sufficient power.**
