# Supplementary material for: Findings on Thoracic Computed Tomography Scans and Respiratory Outcomes in Persons with and without Chronic Obstructive Pulmonary Disease: A Population-Based Cohort Study
Source: PLoS One. 2016 Nov 18;11(11):e0166745. doi: 10.1371/journal.pone.0166745 (PMC5115801; doi:10.1371/journal.pone.0166745)
Supplement: S1 Text — (DOC) [file pone.0166745.s009.doc]

**S1-text**

**Online text supplement**

**Title**: Findings on thoracic computed tomography scans and respiratory outcomes in persons with and without chronic obstructive pulmonary disease: a population-based cohort study.

^1^Wan C Tan, ^2^Cameron J Hague, ^2^Jonathon Leipsic, ^3^Jean Bourbeau,  ^1^Liyun Zhen, ^3^Pei Z Li, ^1^Don D, Sin,  ^1^Harvey O Coxson, ^1^Miranda Kirby, ^1^James C Hogg, ^2^Rekha Raju,

^4^ Jeremy Road, ^5^Denis E O’Donnell, ^6^Francois Maltais,  ^7^Paul Hernandez, ^8^Robert Cowie, ^9^Kenneth R Chapman, ^10^Darcy D Marciniuk, ^4^ J Mark FitzGerald, ^11^Shawn D Aaron, for the Canadian Respiratory Research Network and the CanCOLD Collaborative Research group*.

***Ethic Boards***

Details of the ethical boards :UBC/ PHC Research Ethics Board, P05-006 (Vancouver); Biomedical-C Research Ethics Board, BMC-06-002(Montreal); UHN REB, 06-0421-B (Toronto); Capital Health Research Ethics Board, CDHA-RS/2007-255 (Halifax); Conjoint Health Research Ethics Board, ID21258 (Calgary); DMED-1240-09 ( Kingston); 2009519-01H (Ottawa); Bio-REB09-162(Saskatoon); CER20459 (Quebec City).

***Method: Computed tomographic scans of the lungs.***

Scanning was performed without bronchodilation within one day of lung function testing. All CT scans were acquired using multidetector-row CT scanners with a minimum of 16 rows at suspended full inspiration without administration of intravenous contrast. Exposure settings were 120kVp and 50mAs; images were reconstructed using 1.0mm (Siemens) or 1.25mm (GE) contiguous slices and a high spatial frequency reconstruction algorithm (GE: bone; Siemens: B65f) at a 512 x 512 matrix. All images were displayed on a stand-alone medical imaging workstation (GE Healthcare, Milwaukee, WI), using a standard Window Width of 1000 and a Window Level of -700 HU.

***Grading for emphysema***

For grading emphysema, each lung was divided into 6 zones [(upper-left and upper-right above the carina, mid (middle-left and middle-right) between carina and inferior pulmonary veins and lower ( lower-left and lower-right) zones] and the extent of zonal emphysema scored on a 5 point scale as follows: 0 = no emphysema, 1 = 1-25% (trivial), 2 = 26-50% (mild), 3 = 51-75% (moderate), 4 = 76-100 % (severe-very severe) . The presence of emphysema was a summation emphysema score of 1 or greater. The presence of expiratory air-trapping, bronchial wall thickening and bronchiectasis were assessed based on the morphological criteria from the Fleishner glossary of terms for thoracic imaging.

***Interobserver agreement in a subset of 50 subjects.***

Weighted kappa for The weighted kappa ( 95% CI) ] was considered ‘substantia’l for emphysema [0.58 (0.38, 0.78)], ‘excellent’ for bronchiolitis [0.84 (0.72, 0.97)]; and ‘fair’ for bronchiectasis 0.43(0.06, 0.81), expiratory air-trapping 0.23( 0.049-0.412) and bronchial wall thickening 0.34(0.08,0.61) .
